# The Alberta Newborn Screening Approach for Sickle Cell Disease: The Advantages of Molecular Testing

**DOI:** 10.3390/ijns7040078

**Published:** 2021-11-16

**Authors:** Janet R. Zhou, Ross Ridsdale, Lauren MacNeil, Margaret Lilley, Stephanie Hoang, Susan Christian, Pamela Blumenschein, Vanessa Wolan, Aisha Bruce, Gurpreet Singh, Nicola Wright, Jillian S. Parboosingh, Ryan E. Lamont, Iveta Sosova

**Affiliations:** 1 Newborn Metabolic Screening and Biochemical Genetics Laboratory, University of Alberta Hospital, Alberta Precision Laboratories, Edmonton, AB T6G 2B7, Canada; Janet.Zhou@albertaprecisionlabs.ca (J.R.Z.); Ross.Ridsdale@albertaprecisionlabs.ca (R.R.); Lauren.MacNeil@albertaprecisionlabs.ca (L.M.); Margaret.Lilley@albertaprecisionlabs.ca (M.L.); Stephanie.Hoang@albertaprecisionlabs.ca (S.H.); Susan.Christian@albertaprecisionlabs.ca (S.C.); Pamela.Blumenschein@albertaprecisionlabs.ca (P.B.); Vanessa.Wolan@albertaprecisionlabs.ca (V.W.); 2 Department of Laboratory Medicine and Pathology, University of Alberta, Edmonton, AB T6G 2B7, Canada; 3 Department of Medical Genetics, University of Alberta, Edmonton, AB T6G 2H7, Canada; 4 Department of Pediatrics, University of Alberta, Edmonton, AB T6G 1C9, Canada; Aisha.Bruce@albertahealthservices.ca; 5 Department of Pediatrics, Cumming School of Medicine, University of Calgary, Calgary, AB T3B 6A8, Canada; Gurpreet.Singh@albertahealthservices.ca (G.S.); Nicola.Wright@albertahealthservices.ca (N.W.); 6 Department of Medical Genetics, Cumming School of Medicine, University of Calgary, Calgary, AB T3B 6A8, Canada; Jillian.Parboosingh@albertaprecisionlabs.ca (J.S.P.); Ryan.Lamont@albertaprecisionlabs.ca (R.E.L.); 7 Alberta Children’s Hospital Research Institute for Child and Maternal Health, Cumming School of Medicine, University of Calgary, Calgary, AB T2N 4N1, Canada

**Keywords:** sickle cell disease, sickle cell trait, newborn screening

## Abstract

Sickle cell disease (SCD), a group of inherited red blood cell (RBC) disorders caused by pathogenic variants in the beta-globin gene (*HBB*), can cause lifelong disabilities and/or early mortality. If diagnosed early, preventative measures significantly reduce adverse outcomes related to SCD. In Alberta, Canada, SCD was added to the newborn screening (NBS) panel in April 2019. The primary conditions screened for are sickle cell anemia (HbS/S), HbS/C disease, and HbS/β thalassemia. In this study, we retrospectively analyzed the first 19 months of SCD screening performance, as well as described our approach for screening of infants that have received a red blood cell transfusion prior to collection of NBS specimen. Hemoglobins eluted from dried blood spots were analyzed using the Bio-Rad™ VARIANT nbs analyzer (Bio-Rad Laboratories, Inc., Hercules, CA, USA). Targeted sequencing of *HBB* was performed concurrently in samples from all transfused infants. During the period of this study, 43 of 80,314 screened infants received a positive NBS result for SCD, and of these, 34 were confirmed by diagnostic testing, suggesting a local SCD incidence of 1:2400 births. There were 608 infants with sickle cell trait, resulting in a carrier frequency of 1:130. Over 98% of non-transfused infants received their NBS results within 10 days of age. Most of the 188 transfused infants and 2 infants who received intrauterine transfusions received their final SCD screen results within 21 ± 10 d of birth. Our SCD screening algorithm enables detection of affected newborns on the initial NBS specimen, independent of the reported blood transfusion status.

## 1. Introduction

Hemoglobinopathies comprise a clinically heterogeneous group of blood disorders, characterized by a lack or malfunction of the hemoglobin molecule. Hemoglobin (Hb), the main component of red blood cells, is a tetrameric protein made up of two alpha-like and two beta-like polypeptide chains. Each of the four subunits surrounds an iron-containing heme moiety that can bind one molecule of oxygen. The main function of Hb is to carry oxygen and carbon dioxide through the blood. The globin genes are developmentally regulated such that different alpha- and beta-like globin genes are expressed during different stages of ontogenesis. For much of gestation and at birth the predominant hemoglobin is fetal hemoglobin (HbF), made of two alpha chains and two gamma chains (α_2_γ_2_) [1]. However, during the first year of life the erythroid cells switch to an adult-like pattern with predominant expression of adult hemoglobin (HbA, α_2_β_2_), with about 2% HbA_2_ (α_2_δ_2_) and less than 1% HbF [2].

Hemoglobinopathies fall into two main groups: thalassemia syndromes caused by reduced production of functional hemoglobin, and hemoglobinopathies caused by structural hemoglobin variants. To date, more than 1000 disorders of hemoglobin synthesis and/or structure have been identified and characterized [3]. The most common and medically important structural hemoglobin variants are three beta chain (*HBB*; NM_000518.4) variants: HbS (c.20A>T, p.Glu7Val), HbE (c.79G>A, p.Glu27Lys), and HbC (c.19G>A, p Glu7Lys) [4]. 

Sickle cell disease (SCD) represents a group of autosomal recessive structural hemoglobinopathies caused by pathogenic variants in the β-globin gene, with the presence of at least one HbS variant. Sickle cell anemia (SCA) denotes homozygosity for the HbS variant and is the most common, clinically apparent, and best-studied form of this disease [5]. Other forms of SCD result from coinheritance of HbS with other abnormal β-globin chain variants, the most common forms being sickle-hemoglobin C disease (HbS/C) and two types of sickle β-thalassemia (HbS/β^+^-thalassemia and HbS/β°-thalassemia) [6].

The pathophysiology of SCD is the result of sickling red blood cells (RBCs), which occur when deoxygenated HbS polymerizes within RBCs, shortening the lifespan of RBCs. Consequently, SCD patients often suffer from chronic hemolytic anemia. Sickle cells also become abnormally adhesive, especially to blood vessel endothelium. The resulting microvascular obstruction and impediment of blood flow can lead to ischemia and infarction, causing injury of organs and tissues or even death. Clinically, HbS/C and HbS/β^+^-thalassemia are usually less severe than HbS/S, while HbS/β°-thalassemia manifests similarly to HbS/S [5]. In contrast to the severe manifestation of HbS/S, heterozygous carriers (or sickle cell trait; SCT) are largely asymptomatic; however, they have an increased risk of certain health complications such as chronic kidney disease, renal medullary cancer, hematuria, exertional rhabdomyolysis, and exercise-related sudden death [7,8,9,10]. 

Current treatment options of SCD focus largely on best supportive care, including blood transfusions and pain medication [11]. Hydroxyurea, the first U.S. Food and Drug Administration (FDA) approved drug to treat SCD, prevents polymerization of HbS by increasing the production of HbF by a still unknown mechanism [2,11,12]. Between 2017 and 2019, the FDA approved three additional SCD treatments: L-glutamine and crizanlizumab for preventing acute vaso-occlusive crises in patients 5 years and 16 years of age and older, respectively, as well as voxelotor, an inhibitor of deoxygenated sickle Hb polymerization for patients 12 years of age and older [13]. Hematopoietic stem cell transplantation (HSCT) is currently the only potential cure for SCD [2,4,14]. However, widespread use of HSCT is limited by the lack of suitable donors and risks associated with graft rejection.

Numerous studies provide clear evidence that life-threatening early complications of SCD can be largely avoided if the diagnosis is made early, ideally in the first three to six months of life [15]. Introduction of NBS for SCD has significantly contributed to decreased morbidity and mortality among affected individuals. In the United States, since implementation of universal NBS for SCD, mortality has decreased by 50% in affected children aged 1 to 4 years, and the overall life expectancy has increased from a median of 14.3 years to between 42 and 53 years in males, and between 46 to 58.5 years in females [16,17].

In Alberta, Canada, SCD was added to the newborn screening panel in April 2019 [18]. The primary conditions screened for are sickle cell anemia (HbS/S), HbS/C disease, and HbS/β thalassemia. In this study, we retrospectively analyzed the first 19 months of performance of newborn screening for SCD in Alberta, as well as described our approach for screening of post-RBC-transfused infants.

## 2. Materials and Methods

### 2.1. Study Design

We conducted a retrospective cohort study of all infants born and registered in Alberta between 1 April 2019 and 31 October 2020, and screened for SCD by the provincial Newborn Metabolic Screening (NMS) Laboratory in Edmonton. Screened infants who were not registered in Alberta were excluded from this study.

### 2.2. Newborn Screening Specimen Collection

Newborn blood spot screening is offered to the parents/guardians of all infants registered with the Alberta government. Newborn screening is voluntary, and consent is verbal [18]. Collection of the blood specimens is recommended to occur between 24 and 72 h of age, ideally closer to 24 h. If a newborn requires an RBC transfusion (RBCT) within the first 24 h of life, our recommendation is to collect a sample before the transfusion, followed by a second, post-transfused collection at the age equal or greater than 24 h. Dried blood spot (DBS) specimens are collected as previously described [18]. Briefly, capillary blood spots obtained by heel-stick are collected on Whatman 903 filter paper attached to the newborn screening requisition. Samples are then air-dried for a minimum of three hours and transported at ambient temperature in a sealed newborn screening envelope to the provincial screening laboratory in Edmonton. Since some conditions may be missed in low birth weight (BW) infants when screened early after birth, all infants with BW less than 2000 g are recommended to have blood spots recollected at 21–28 days of age, even if the initial screen result was normal.

### 2.3. Hemoglobin Pattern Analysis

Hemoglobins eluted from a single 3.2 mm dried blood disk with 250 µL of deionized water in a 96-well microplate are analyzed by cation exchange high-performance liquid chromatography (HPLC) using the Bio-Rad™ VARIANT nbs analyzer (Bio-Rad Laboratories, Inc., Hercules, CA, USA) with the corresponding Bio-Rad VARIANT™ NBS Sickle Cell Program kit, in full compliance with the manufacturer’s instructions. This system provides a rapid qualitative screen, and is intended to detect hemoglobins F, A, S, C, D, and E. Quality control samples containing known amounts of F, A, S, C, D, and E hemoglobin variants with characterized retention times are analyzed with each run. Hemoglobins are eluted from the column based on their positive charge, and are reported in descending order of quantity, expressed as percentage of total peak area (Area %). The stability of DBS hemoglobins, as determined by our NBS laboratory, is up to 2 weeks after collection.

A normal hemoglobin profile in full-term infants at birth is represented by approximately 80% of hemoglobin F and 20% of hemoglobin A [19,20]. HbF has weaker positive charge than HbA, and is eluted before HbA. HbF is partially acetylated, and the less positively charged acetylated fraction (HbF1) elutes before the non-acetylated HbF. HbE and HbA_2_ coelute and cannot be distinguished with this method. However, at birth HbA_2_ is usually below detection levels and the peak likely represents HbE, unless the infant was transfused. The absence of HbA most likely represents beta-thalassemia major (β°-thalassemia, i.e., lack or almost complete lack of beta chains), although the absence of HbA can also be caused by homozygosity for deletional hereditary persistence of fetal hemoglobin (HPFH; very rare) or a β°thalassemia/HPFH combination [21]. FS pattern can represent homozygous sickle cell disease (HbS/S), HbS/β°thalassemia, or sickle hereditary persistence of fetal hemoglobin (HbS/HPFH). The HPLC method can also detect alpha-thalassemia and alpha–thalassemia carriers. Excess of gamma chains in these patients can be detected at birth by the presence of Hb Bart’s peak, (γ_4_ homotetramer) and to some extent, the area of Hb Bart’s peak correlates with the number of deleted/inactivated α-globin genes. In HbH disease with three defective α-genes (−/−α), the area of the Bart’s peak represents approximately 22.5–25.0% (or more) of the total area [21,22]. Hb Bart’s, if present, elutes at the beginning of a run as a sharp, narrow peak (within the FAST peak retention time). However, it coelutes with bilirubin which can be mistaken for Hb Bart’s on Hb HPLC (with the Bio-Rad™ VARIANT nbs instrument both Hb Bart’s and bilirubin have retention times around 0.1 min). Furthermore, degradation of hemoglobin can also cause a high but broad FAST peak with an elevated baseline. 

### 2.4. SCD Screening Algorithm

**Figure 1:**
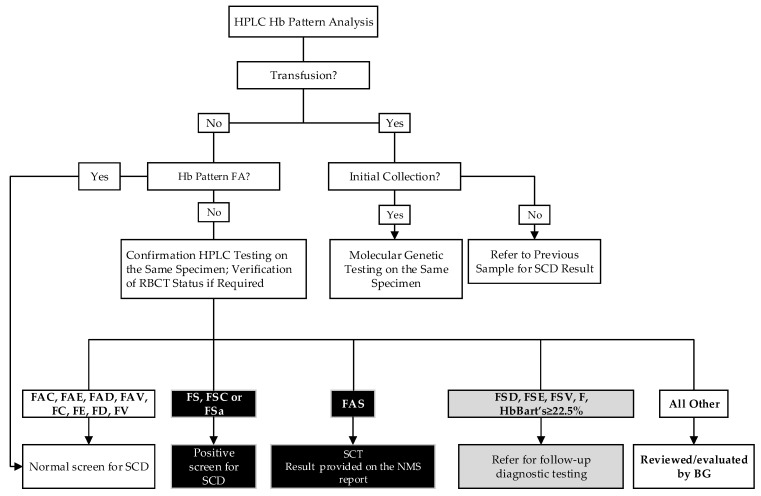
Alberta Sickle Cell Disease Screening Algorithm. V = unknown variant. i.e., any hemoglobin for which a quality control is not in use, except for Hb Bart’s; FSa = HbS/β thalassemia; BG, biochemical geneticist.

The screening algorithm is summarized in Figure 1. Briefly, in non-transfused infants, if the initial HPLC hemoglobin pattern is FA (with HbA peak less than 30 or 35% in preterm or full-term infants, respectively), the screening for SCD is complete and the result is released as “Normal SCD screen result”. If the hemoglobin pattern on the initial punch is abnormal (i.e., other than FA), the specimen is repunched and reanalyzed on the following business day. In addition to FA, homozygosity for non-sickle variants and heterozygosity for HbC, HbD, HbE, and HbV (unknown variant, i.e., any hemoglobin for which a quality control is not in use) are reported as normal screen results. Apart from the three primary sickling conditions, we report other sickling and/or clinically significant hemoglobinopathies detected (Figure 1). 

Concurrent to HPLC analysis, molecular genetic screening is performed in all newborns who either received a red blood cell (RBC) transfusion prior to the first NBS specimen collection, or if the initial hemoglobin pattern is highly suggestive of RBC transfusion (i.e., AF pattern in a newborn). If the RBC transfusion was not indicated on the requisition form, and the FA pattern shows HbA peak greater than 30% or 35% in a preterm or a full-term infant, respectively, the transfusion status is reviewed and/or verified, and molecular testing may be performed. The molecular testing is usually completed within 7–21 days. To avoid a delay in the reporting for other NBS conditions in these infants, the NBS report is released once the screening for the remaining 20 conditions is completed with a “Pending SCD results” comment, and the final report is issued when the molecular testing is completed. If a transfused infant had an additional newborn screening specimen collected before the RBC transfusion, or the molecular testing had already been performed on a previously collected specimen, the HPLC screen results are released with an interpretive comment: “Refer to previous sample for Sickle Cell Disease result.”

### 2.5. Molecular Genetic Testing

The DNA is extracted from a single 3.2 mm dried blood disk and targeted sequencing of the *HBB* gene is performed to identify variants responsible for HbS: c.20A>T (p.Glu7Val) and HbC: c.19G>A (p.Glu7Lys), using the cDNA transcript NM_000518.4 as a reference (primer sequences available upon request). PCR amplification and sequencing are performed followed by capillary electrophoresis. Subtraction analysis software (SEQPATIENT, Version 4.4.0, Build 509, J.S.I Medical Systems GmbH, Ettenheim, Germany) is used to identify sequence variation. This assay will not detect other rare variants outside the sequenced region or exonic/whole gene deletions. Thus, this molecular testing cannot exclude the possibility of Hb S/β thalassemia in an HbS carrier (SCT) and cannot distinguish between homozygosity for HbS or HbC and the compound heterozygous states of HbS or HbC with beta thalassemia.

### 2.6. Calling Out Abnormal Screening Results 

All abnormal SCD results are reported to the ordering healthcare providers by a genetic counsellor who explains the result. A preliminary NBS report and a SCD information sheet are faxed to the ordering provider to support their NBS result disclosure to the family. Results are also communicated to one of the two pediatric hematology clinics in the province, depending on the infant’s location. Confirmatory diagnostic testing is arranged by the hematologist, results are communicated to the families, and clinical follow up is arranged, as indicated, by the hematology clinic.

When a newborn is identified to have SCT, the result is provided on the NBS report. In addition, the ordering provider is faxed a letter and a SCT information sheet which includes testing and assessment algorithm for the newborn, their parents, and their siblings [9].

## 3. Results

### 3.1. Screened Infants

Between 1 April 2019 and 31 October 2020, a total of 80,761 infants were born and registered in Alberta, and of these, 80,314 (99.45%) received NBS (Alberta Health Newborn Metabolic Screening Application 2019–2020, data extracted on 12 January 2021). GA and transfusion status of the screened infants are shown in Table 1.

### 3.2. Screening of Transfused Infants 

A total of 188 newborns received an RBCT prior to the initial NBS specimen collection. Of those, 86 (45.7%) were preterm infants (Table 1). Excluding two infants with an unsatisfactory initial NBS specimen who died before the recollection, all transfused infants received their final NBS SCD result within 21 ± 10 d of birth. All screened transfused infants had normal SCD results. NBS also detected two preterm infants who received intrauterine transfusion (IUT). In both cases the transfusion was not indicated on the NBS requisition; however, the initial HPLC pattern showed high HbA peak area. Following communication with the care provider, the IUT was confirmed. The first infant was male with birth weight (BW) 3110 g, born at GA 36 w with an IUT at GA 32w6d. His FA pattern showed F1 (8.6%), F (23.1%), A (36.1%), and E/A2 (1.4%). The second infant, male and born at GA 35 w (BW 2480 g) who received an IUT at 31w2d, had an abnormal AF pattern showing F1 (8.6%), F (23.1%), and 56.8% for A. Molecular genetic testing revealed a normal screen result in the first IUT infant, and SCT in the second infant (the second infant is described with more details in Section 3.5, Infant FP1). 

In total, 241 specimens were sent for molecular genetic testing during the period of this study.

### 3.3. Positive Screening Results for SCD 

During the first 19 months of screening for SCD, the NMS Laboratory in Edmonton reported 43 positive screens for SCD, 3 positive screens for other hemoglobinopathies, and 608 carriers of HbS. A summary of the screening results and diagnostic outcomes of the positive screens are provided in Table 2. Screening correctly identified 35 infants with SCD, 34/35 having one of the target conditions and 1 infant with HbS/HPFH.

### 3.4. Hemoglobin Variant Carriers 

The carriers of different hemoglobin variants are summarized in Table 2. Only HbS carriers are reported on the newborn screen report.

### 3.5. False Positive Screening Results

Eight infants had false positive results (infants FP1–FP8; Table 3). FP1 infant was a preterm infant (born at GA 36 w) who received an IUT at 32w6d. The molecular genetic analysis identified one HbS variant. However, due to the limitation of the molecular screening test to detect whole gene deletions, the screen result was issued as a positive screen for SCD. Diagnostic follow-up testing confirmed the SCT in this infant.

Infant FP2 had an abnormal HPLC Hb pattern showing four peaks (FSA5; F = 59.1%; S = 11.6%; A = 7.6%; an unknown peak 5 (2.0%) eluting between S and C retention time windows). The diagnostic molecular testing revealed heterozygosity for an Hb alpha-2 gene variant (Hb Q-Iran). Diagnostic evaluation of infant FP3, also with an FSA5 profile, is still in progress. However, the biochemical investigation was suggestive of an unknown alpha chain variant. In infant FP4 (hemoglobin pattern FA with an abnormally shaped A peak (24.3%) and a very small S peak (2.0%)), the molecular genetic testing did not detect any reportable *HBB* variant. However, follow-up biochemical testing was suggestive of an unknown beta chain variant. Infant FP5 had an FAS pattern with a large unknown peak (18.8%) that eluted between the F and A retention time windows. This infant was referred for diagnostic testing, which confirmed the SCT. Infants FP6–FP8 had FSA patterns and were all confirmed to have SCT (FAS). 

## 4. Discussion

SCD is among the most common genetic disorders in the world, affecting over 300,000 newborns annually with estimates for further increases to over 400,000 annual births within the next generation [23,24,25]. SCD is the most common hemoglobinopathy in Africa, the Middle East, and India. However, in recent years its incidence has increased dramatically in Europe and North America because of the high rate of migration of people from endemic areas [26]. 

In April 2019, the province of Alberta joined British Columbia, Ontario, Quebec, Nova Scotia, Prince Edward Island, and New Brunswick in screening for SCD, increasing the number of Canadian provinces currently screening for SCD to seven. Infants born in the Canadian territories Yukon, Northwest Territories, and Nunavut are screened by the provincial NBS laboratories in British Columbia, Alberta, and Ontario, respectively. 

NBS in Alberta is voluntary, but the participation rate is high [18]. During the duration of this study, 99.45% of all registered infants were screened. We identified 34/80,314 infants with SCD (Table 2), suggesting a local birth incidence in Alberta of approximately 1:2350. The birth incidence of SCA (HbS/S) was approximately 1:3350, and the birth incidence of HbS/C was 1:8900. Therrell et al. reported an overall SCD annual birth incidence of 1:1941 across the USA [27]. In Canada, annual birth incidence rates of SCD in provinces with large multiethnic populations such as Ontario and Quebec are comparable to those in the USA. Similar to the USA, the estimated annual birth incidence varies geographically with reported estimates of 1:17,721 in British Columbia, 1:5650 in Ontario, and 1:1852 in Quebec [16,28]. In Europe, the pooled birth incidence of SCA is 1:2300, although, also in Europe, this varies between countries [29]. The local birth incidence of the primary target SCD in Alberta appears to be 7.5-fold higher than the annual birth incidence in the neighboring province of British Columbia, and 2.4-fold higher than in Ontario. However, it is comparable to the SCD annual birth incidence in Quebec, USA, and Europe. Differences in birth incidence are likely due to differences in ethnic origins of the populations; however, we are unable to comment on the ethnic origin of our positive screens since this information is not collected on the Alberta NBS requisition.

The implementation of NBS for SCD has significantly improved the quality of life of the affected babies. Their parents have more time to adjust to a difficult diagnosis, understand the concept of hemoglobin F, and more willingly embrace hydroxyurea. Monagel et al. reported that prior to the NBS implementation, the mean age of SCA diagnosis for the patients born in Alberta who attended Alberta Children’s Hospital hematology clinic from January 2003 to January 2014 was 21.4 ± 21 months [30]. Thanks to the introduction of the NBS for SCD, the pediatric hematologists and pediatricians (or most responsible practitioners for the infant) are informed about the positive screen within 72 h of final testing and infants are seen by a pediatric hematologist within a week to initiate confirmatory testing. All infants with a confirmed diagnosis of SCD are started on penicillin/amoxicillin prophylaxis by two months of age and given a letter approving immunizations for functional asplenia. As well, education including signs and symptoms of anemia and sepsis, splenic palpation technique, signs and symptoms of dactylitis, and program contact information is provided. Additional support and counselling are also provided to the parent(s) regarding the probable diagnosis and inheritance patterns. Testing of parents and siblings is also offered to the family. All infants confirmed to have SCD are followed in the comprehensive Sickle Cell Disease Clinic which follows the Canadian Hemoglobinopathy Association Sickle Cell Disease Consensus Statement (https://www.canhaem.org/scd-consensus-statement/; accessed on 4 October 2021) and National Heart, Lung, and Blood Institute Clinical Guidelines (https://www.nhlbi.nih.gov/health-topics/evidence-based-management-sickle-cell-disease; accessed on 4 October 2021).

During the period of this study, 8 of the 46 newborns with positive screen were confirmed to be false positives (FP; Table 3). The lowest concordance rates for screening and confirmatory testing occurred for the results of FSA, a pattern that may indicate a SCT or HbS/β thalassemia. For the distinction between FSA (HbS/β^+^ thalassemia or HbS/β^++^ thalassemia) and an HbS carrier, the relative quantification of HbA and HbS and the ratio between HbA/HbS are used. However, this approach bears the risk of false positive and false negative results as there is an overlap between HbS/β^+^ thalassemia or HbS/β^++^ thalassemia and a simple carrier state for HbS, especially in premature babies [15]. In our routine interpretation we use a cutoff of HbS/HbA = 2 to distinguish infants with severe HbS/β^+^ thalassemia (i.e., FSa). If the HbS/HbA ratio lies between 1 and 2, the interpretation of the FSA pattern is more challenging. Since the consequences of not identifying a child with SCD are much greater than misidentifying a newborn with SCD who is later found to have SCT, the preference is to refer these infants for diagnostic testing. Follow-up diagnostic testing revealed that all three infants who screened positive for FSA on HPLC had S trait only (SCT). 

An additional subgroup of FP screens represents screens with Hb patterns containing variants coeluting within the S retention window. A recent search in the hemoglobin variant database (http://globin.cse.psu.edu, accessed on 10 September 2021) showed that there are currently more than 1400 known variants. About thirty different variants, including several alpha-chain variants, have been identified to have a retention time that overlaps with the retention time range of HbS on HPLC [31,32]. Two of our false positive screens (FP2 and FP3, Table 3) were due to elution of alpha-chain variants within the HbS window.

One FP result was due to the limitations of the molecular method we use to screen transfused infants. Since it cannot detect whole gene deletions, all screens with one S pathogenic variant detected are issued as positive screens for SCD (i.e., HbS/β^0^ thalassemia) and further confirmatory testing is required to distinguish SCT and SCD in these infants. 

Screening of preterm infants represents another challenge. Current medical and technological advances have significantly improved the survival of preterm infants worldwide. In our study, 5971 (7.44%) of all screened infants were preterm. Of these, 86 were transfused before the newborn screening sample collection. Preterm infants have an increased likelihood of false positive screening results due to low levels or absence of HbA [33]. During the period of this study, 261 infants were born at GA less than 28 w, and 35 of these extremely preterm infants received an RBCT prior to NBS sample collection (Table 1). In our study, the preterm infant with the lowest GA was a transfused infant born at GA 22 w (BW 580 g). The infant with the lowest BW was a 385 g infant (GA 23 w) with a normal HPLC Hb pattern for the GA (80.7% F; 4.6% A). In total, eight infants had BW less than 500 g, and three of those received RBC transfusion prior to the NBS. All preterm infants, excluding the FP1 infant described in Section 3.5, had a normal screen for SCD.

After the announcement of the expansion of the newborn screening panel by the Alberta Government in February 2018, two multidisciplinary working groups were formed to create the disclosure policy for infants with SCT and the report policy for other hemoglobinopathies detectable by HPLC. Alberta Health chose to implement a universal disclosure policy for SCT [9] and include SCT results in the final newborn screen result. Several clinical and epidemiological studies have shown that heterozygosity for HbS may contribute to specific disease processes, particularly under extreme conditions that promote HbS polymerization [7]. During the period of this study, about 1 in 132 Alberta babies were born with SCT (Table 2). Assuming Hardy–Weinberg equilibrium (HWE), the predicted HbS allele frequency should be 1:29; however, deviations from HWE are common in surveys of hemoglobin variants in the study regions. The two primary factors traditionally assumed to account for significant deviations from HWE are inbreeding or consanguinity and stratification due to short- and/or long-distance migration [34]. These two factors likely also account for the differences in the predicted vs. observed HbS allele frequency in Alberta. To date, we have not been informed of any missed SCD cases.

For newborns found to be carriers of an Hb variant other than HbS, the NBS result is issued as normal. In regard to reporting other hemoglobinopathies, the pre-implementation working groups came to a consensus which is summarized in Figure 1. 

**Figure 2:**
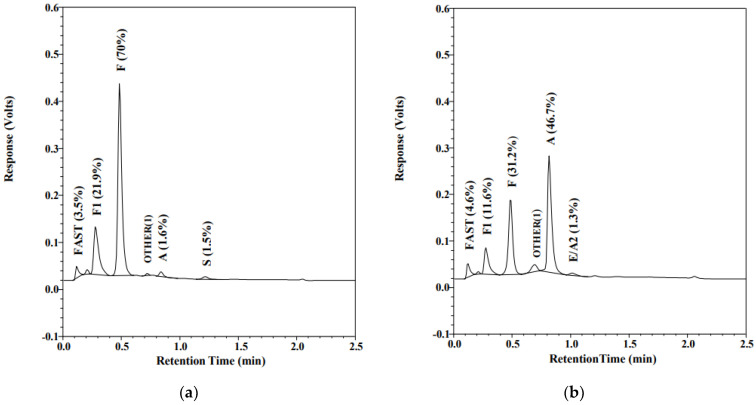
Chromatogram of HPLC (VariantTM newborn screening (nbs), Bio-Rad Laboratories, Europe). Percentage of total area for each peak is displayed in the parenthesis: (**a**) Hb profile on a preterm (GA = 25 w, BW = 804 g), non-transfused 24-hour-old infant; (**b**) infant’s profile at the age of 21 days, 5 days after red blood cell transfusion.

Over 98% of registered, non-transfused infants have their screening results reported within 10 days of age. Most transfused infants receive their final SCD screen results within the first month of life. When screening for SCD, transfused infants represent a special challenge. RBC transfusions independently suppress endogenous erythropoiesis by raising oxygen-carrying capacity. In an affected infant, reduced erythropoiesis leads to less detectable or undetectable HbS. In addition, transfusions also complicate screening by supplying exogenous HbA [35]. Most NBS screening programs require a repeat screening to be performed 120 days after the last transfusion; however, there are several cases of late or missed diagnoses of SCD due to non-compliance with the four-month recollection requirement. Similar to at least one other program [36,37], Alberta implemented concurrent molecular genetic testing on all transfused infants to enable identification of SCD in these infants on the initial collection, without the requirement of the 120-day post-last-transfusion recollection. Figure 2 illustrates how RBCT can mask the newborn’s SCT phenotype. Figure 2a shows an FAS pattern on a non-transfused preterm infant (GA 25 w, BW 840 g). The initial sample was collected at 24 h of life and the screen result was reported as SCT. As per the protocol for infants weighing less than 2000 g, a second NBS specimen was collected at 21 days of age; however, this infant had been transfused at the age of 16 days. The pattern in Figure 2b shows a typical post-transfusion AF pattern. The RBCT masked the SCT; the S peak was undetectable after transfusion. *HBB* sequence analysis performed on both the pre- and post-transfusion specimens detected heterozygosity for HbS (*HBB* c.20A>T) in both specimens. Molecular genetic testing detects the HbS and HbC variants, independently of the transfusion status, eliminating the risk of a missed or delayed SCD diagnosis in transfused newborns. 

## 5. Conclusions

In Alberta, NBS for SCD was successfully implemented in April 2019 with the following primary target conditions: HbSS, HbSC, and HbS/β thalassemia. SCT results are also disclosed in the final newborn screen report. Transfused infants undergo a two-step screening, with the second-step screen being targeted sequencing analysis of *HBB* gene to identify HbS and HbC variants only. 

During the first 19 months of screening for SCD, we identified 34 infants having the primary target condition, suggesting a local birth incidence in Alberta of approximately 1:2400 births. Additionally, four infants with other hemoglobinopathies and 608 infants with SCT were detected. The current screening algorithm for SCD enables detection of affected newborns shortly after birth, independent of the reported blood transfusion status, reducing the risk of missed or delayed SCD diagnoses. Early implementation of preventive care has significantly improved the quality of life of these infants and their families.

## Figures and Tables

**Table 1:** Gestational age and transfusion status of Alberta infants born between 1 April 2019 and 31 October 2020 and screened in the NMS Laboratory in Edmonton.

Screened Infants	*n*	RBCT
Full-term	74,343	102
Preterm	5971	86
Late Preterm 34 to <37 w	4416	24
Moderate Preterm 32 to <34 w	751	11
Very preterm 28 to <32 w	543	16
Extremely Preterm <28 w	261	35
Total	80,314	188 *

* This number does not include two infants with intrauterine transfusion; RBCT, red blood cell transfusion.

**Table 2:** Hemoglobinopathies and variant carriers identified by the NMS Laboratory in Edmonton between 1 April 2019 and 31 October 2020.

Condition *	*n*	Rate per 10,000 Newborns Screened
HbSS	24	3.0
HbSC	9	1.1
HbS/β thalassemia	1	0.1
HbS/HPFH	1	0.1
HbEE	1	0.1
β thalassemia major	2	0.2
Carriers of HbS (FAS)	608	75.7
Carriers of HbE (FAE)	227	28.3
Carriers of HbC (FAC)	197	24.5
Carriers of HbD (FAD)	161	20.0
Carrier of HbQ-Iran	1	0.1
Carrier of HbV (FAV)	1	0.1

* Hemoglobins are reported in descending order of quantity; Hb, hemoglobin; S, HbS; C, HbC; E, HbE; F, fetal hemoglobin; A, adult hemoglobin; V, unknown variant; HPFH, hereditary persistence of fetal hemoglobin; HbSS, sickle cell anemia; HbSC, sickle-hemoglobin C disease; HbEE, hemoglobin E disease.

**Table 3:** False positive screens.

Infant	NBS Result *	Gender	GA	Transfusion	Confirmatory Testing Result
FP1	FAS	M	36 w	IUT	Carrier of HbS (FAS)
FP2	FSA5	M	FT	No	Alpha-2 globin gene variant (HbQ-Iran)
FP3	FSA5	M	FT	No	Unknown alpha chain variant
FP4	FA	F	FT	No	Unknown beta chain variant
FP5	FAS	F	FT	No	Carrier of HbS (FAS)
FP6	FSA	M	FT	No	Carrier of HbS (FAS)
FP7	FSA	F	FT	No	Carrier of HbS (FAS)
FP8	FSA	M	FT	No	Carrier of HbS (FAS)

* Hemoglobins are reported in descending order of quantity; A, adult hemoglobin; F, fetal hemoglobin; S, HbS; FAS, carrier of HbS; FSA, pattern suggestive of HbS/β^+^ thalassemia or HbS/β^++^ thalassemia; FP, false positive; FT, full-term; GA, gestational age; Hb, hemoglobin; IUT, intrauterine blood transfusion; w, week; Peak 5, unknown peak eluting between HbS and HbC retention time windows.

## Abbreviations

α	Alpha
β	Beta
γ	Gamma
BG	Biochemical geneticist
BW	Birth weight
d	Day
DBS	Dried blood spot
FP	False positive
FSa	HbS/*beta* thalassemia
GA	Gestational age
Hb	Hemoglobin
HbA	Adult hemoglobin
HbF	Fetal hemoglobin
HPFH	Hereditary persistence of fetal hemoglobin
HPLC	High-performance liquid chromatography
HSCT	Hematopoietic stem cell transplantation
IUT	Intrauterine transfusion
NBS	Newborn screening
NMS	Newborn metabolic screening
PCR	Polymerase chain reaction
RBC	Red blood cells
RBCT	Red blood cell transfusion
SCA	Sickle cell anemia
SCD	Sickle cell disease
TP	True positive
w	Week
